# Forensische Aspekte der Verordnung medizinischen Cannabis in der Psychiatrie

**DOI:** 10.1007/s00115-023-01529-w

**Published:** 2023-08-14

**Authors:** Anne Koopmann, Harald Dreßing

**Affiliations:** 1 Klinik für Abhängiges Verhalten und Suchtmedizin, Zentralinstitut für Seelische Gesundheit Mannheim, Universität Heidelberg, J 5, 68159 Mannheim, Deutschland; 2 Feuerlein Centrum für Translationale Suchtmedizin (FCTS), Universität Heidelberg, Heidelberg, Deutschland; 3 Klinik für Psychiatrie und Psychotherapie, Zentralinstitut für Seelische Gesundheit Mannheim, Universität Heidelberg, Mannheim, Deutschland

## Rechtliche Rahmenbedingungen

Medizinische Cannabinoide können seit 01.03.2017 von Ärzten jeder Fachrichtung, mit Ausnahme von Zahn- und Tierärzten, verordnet werden. Eine besondere Qualifikation ist nicht erforderlich. Die Verordnung erfolgt über ein Betäubungsmittelrezept. Soll eine Kostenübernahme durch die gesetzliche Krankenkasse erfolgen, muss vor Beginn der Behandlung im individuellen Patientenfall ein Antrag auf Kostenübernahme an die Krankenkasse gestellt werden, der vom Medizinischen Dienst (MD) geprüft wird. Eine Prüfung durch den MD kann jedoch umgangen werden, wenn die Verordnung der medizinischen Cannabinoide auf Privatrezept mit dem Patienten als Selbstzahler erfolgt. Medizinische Cannabinoide enthalten ähnlich wie das auf dem Schwarzmarkt verkaufte Cannabis die Wirkstoffe Tetrahydrocannabinol (THC) und Cannabindiol (CBD). Abhängig von der Sorte variieren in den Präparaten die enthaltenen Anteile des THC bzw. des CBD. Die Höchstmenge Cannabisblüten, die der Arzt innerhalb von 30 Tagen für einen Patienten verordnen darf, beträgt gemäß der Arbeitsgemeinschaft deutscher Apothekenkammern 100.000 mg (100 g; [2]).

## Aktuelle Versorgungssituation

Motiviert durch positive Einzelfallberichte in Internetforen oder aus dem persönlichen Bekanntenkreis sowie positive eigene Konsumerfahrungen, stellen sich in den letzten Jahren zunehmend PatientInnen mit dem Wunsch nach einer Behandlung ihrer psychischen Erkrankung mit medizinischen Cannabinoiden in psychiatrischen Praxen und Institutsambulanzen vor. Diese PatientInnen weisen teilweise lange und komplexe psychiatrische Krankengeschichten mit mehreren gescheiterten medikamentösen und psychotherapeutischen Behandlungsversuchen auf, teilweise leiden sie komorbid an Abhängigkeitserkrankungen. Daher gestaltet sich die Entscheidungsfindung für oder gegen eine Verordnung medizinischer Cannabinoide an die betroffenen PatientInnen für die behandelnden PsychiaterInnen nicht immer einfach. Erfolgen zu sorglose Verordnungen medizinischer Cannabinoide bei unklarer Indikation und insbesondere über die zugelassene Höchstdosis hinaus und wird dies den Ordnungsbehörden bekannt, kann es zu berufs- und strafrechtlichen Verfahren führen, in denen sich psychiatrische GutachterInnen mit komplexen, teilweise nicht leicht zu beantwortenden Fragestellungen zu Indikation und der Verhältnismäßigkeit der Verordnung im Einzelfall auseinandersetzen müssen.

## Fallbeispiel

Anhand einer fiktiven Fallvignette, die unterschiedliche Aspekte aus der gutachtlichen Praxis integriert, sollen diesbezüglich wichtige Aspekte diskutiert werden, wenn an GutachterInnen die Frage gerichtet wird, ob die Verordnung medizinischer Cannabinoide am oder im menschlichen Körper der PatientInnen begründet und ob die Art und die Menge der verschriebenen medizinischen Cannabinoide bei den PatientInnen indiziert waren.

Ein niedergelassener Nervenarzt verordnet einem seiner Patienten während einer mehrjährigen Behandlungszeit in unregelmäßigen, aber oft sehr kurzen Zeitabständen von wenigen Tagen medizinische Cannabinoide unterschiedlicher Sorten mit unterschiedlichem THC-Gehalt, unterschiedlicher Darreichungsformen und ohne Angabe einer Dosierungsempfehlung auf Privatrezept mit dem Patienten als Selbstzahler. Die Diagnosen wechseln im Laufe der Behandlung. Diagnostiziert werden eine generalisierte Angststörung (F41.1), eine Zwangsstörung mit Zwangsgedanken und -handlungen gemischt (F42.2), eine Anpassungsstörung (F43.2) und eine einfache Aktivitäts- und Aufmerksamkeitsstörung (F90.0). Ausführliche psychopathologische Befunde oder testpsychologische Untersuchungen, die diese Diagnosen begründen, finden sich ebenso wenig, wie wissenschaftliche Begründungen für die Verordnung von Cannabinoiden.

## Gutachtliche Einordnung

Die Wirksamkeit medizinischer Cannabinoide wurde für eine Vielzahl von Indikationen in klinischen Studien geprüft. Diese Studien wiesen fast alle (teilweise deutliche) methodische Mängel auf (kleine Studienpopulationen, nicht vorhandene oder schlecht gewählte Kontrollbedingungen, gegen die die Therapie mit medizinischen Cannabinoiden getestet wurde; [4]). So zeigte beispielsweise eine 2019 veröffentlichte Übersichtsarbeit von 31 Studien zum Einsatz medizinischer Cannabinoide bei Angsterkrankungen bzw. Angstzuständen, dass nur 17 die wissenschaftlichen Mindestkriterien erfüllten [1]. Deren Auswertung ergab, dass medizinische Cannabinoide Angstsymptome lediglich bei Personen verbesserten, die zu dem untersuchten Zeitpunkt unter chronischen Schmerzen oder Multipler Sklerose litten. Bei alleinigen Angsterkrankungen scheinen die positiven Effekte der Therapie mit medizinischen Cannabinoiden jedoch nicht vorhanden zu sein [1]. Für das psychiatrische Fachgebiet kann basierend auf der aktuellen Studienlage für keine Indikation eine Behandlungsempfehlung mit medizinischem Cannabis ausgesprochen werden, am besten ist die Datenlage noch zum Einsatz medizinischer Cannabinoide bei einer posttraumatischen Belastungsstörung (F43.1). Beim Vorliegen dieser Diagnose kann nach Ausschöpfen aller anderen medikamentösen und psychotherapeutischen Behandlungsmöglichkeiten ein individueller Heilversuch mit medizinischen Cannabinoiden unternommen werden [4].

Dennoch werden medizinische Cannabinoide gemäß dem Abschlussbericht der Begleiterhebung zur „Verschreibung und Anwendung von Cannabisarzneimitteln“ des Bundesministeriums für Arzneimittel und Medizinprodukte im psychiatrischen Fachgebiet in Deutschland nach Genehmigung durch die gesetzlichen Krankenkassen verhältnismäßig häufig (sechsthäufigste Verordnungen im Vergleich mit allen anderen medizinischen Fachgebieten) verschrieben [3]. Es steht zu vermuten, dass eine weitaus größere Anzahl an Verordnungen über Privatrezepte mit dem Patienten als Selbstzahler hinzukommen, die bisher in keiner Statistik erfasst werden und deren Indikation und Verhältnismäßigkeit aus forensischer Sicht nicht immer einfach beurteilt werden kann.

Die Konstellation, wie sie in der Fallvignette dargestellt ist, kommt nach persönlicher Erfahrung der Autoren im psychiatrisch-psychotherapeutischen Versorgungsalltag häufiger vor, führt aber nur in seltenen Fällen zu berufs- oder strafrechtlichen Verfahren mit nachfolgender Begutachtung durch KollegInnen. Dennoch sollten sich ÄrztInnen bewusst sein, welche Implikationen die nicht sachgerechte Verordnung von Cannabinoiden haben kann. Hier ist in der Versorgungspraxis oft auch ein starkes Drängen von PatientInnen zu beobachten, die auf ihr vermeintliches Recht pochen, diese „Medizin“ verordnet zu bekommen. Berichte in den Medien über vermeintliche Therapieerfolge wecken hier teilweise Erwartungen, die durch evidenzbasierte Studien nicht gedeckt sind. Es ist deshalb wichtig, dass PsychiaterInnen über die Studienlage informiert sind und mit entsprechenden sachlichen Argumenten ihre Position vertreten können und sich nicht auf eine „slippery slope“ begeben, auch wenn dies dazu führen kann, gewisse PatientInnen zu verlieren.

In der Konstellation der fiktiven Fallvignette sprachen, neben der dargestellten fehlenden wissenschaftlichen Evidenz für den Einsatz medizinischer Cannabinoide bei den vergebenen Diagnosen, die wiederholten Verordnungen medizinischer Cannabinoide mit sehr geringen zeitlichen Abständen und die Verordnung wechselnder Darreichungsformen mit einem unterschiedlichen THC-Gehalt dagegen, dass diese im menschlichen Körper des Patienten begründet und die Art und die Menge der verschriebenen medizinischen Cannabinoide bei dem Patienten indiziert waren. Aus dieser gutachtlichen Einschätzung können sich für verordnende ÄrztInnen erhebliche berufs- und strafrechtliche Konsequenzen ergeben.

## Schlussfolgerungen

Vor dem Hintergrund, dass Fallkonstellationen denkbar sind, in denen die forensische Einordnung der Verhältnismäßigkeit der Verordnungen medizinischer Cannabinoide im Einzelfall schwieriger ist, wäre aus unserer Sicht die Durchführung kontrollierter, randomisierter Studien mit einer ausreichend großen Zahl an Teilnehmenden getrennt nach unterschiedlichen psychiatrischen Diagnosegruppen dringend erforderlich, um die Wirksamkeit medizinischer Cannabinoide bei den einzelnen psychiatrischen Krankheitsentitäten besser einordnen und in der Versorgungspraxis vor Therapiebeginn eine bessere Indikationsstellung vornehmen zu können.

## Fazit für die Praxis

- Vor Verordnung medizinischer Cannabinoide sollte eine leitliniengerechte Diagnostik erfolgen.
- Vor Verordnung medizinischer Cannabinoide sollte die wissenschaftliche Evidenz für die spezifische Indikation geprüft werden.
- Bei der Verordnung medizinischer Cannabinoide sollten die zulässigen Höchstdosen pro Verschreibungsintervall beachtet werden.
- Während der Verordnung medizinischer Cannabinoide sollten regelmäßig Maßnahmen erfolgen, die einen Missbrauch der Substanz verhindern.
